# Bezold’s abscess secondary to os tympanicum cholesteatoma in Goldenhar syndrome

**DOI:** 10.1259/bjrcr.20200121

**Published:** 2021-03-26

**Authors:** Matteo Minerva, Silvia Valeggia, Stefano Fusetti, Elisabetta Zanoletti, Renzo Manara, Davide Brotto

**Affiliations:** 1 Department of Medicine - DIMED, Radiology Institute, University of Padova, Azienda Ospedale - Università Padova, Padova, Italy; 2 Maxillo-Facial Surgery Unit, Neurosciences Department, University of Padova, Azienda Ospedale - Università Padova, Padova, Italy; 3 Department of Otorhinolaryngology Section, Neurosciences, University of Padova, Azienda Ospedale - Università Padova, Padova, Italy; 4 Neuroradiology Unit, Neurosciences Department, University of Padova, Azienda Ospedale - Università Padova, Padova, Italy

## Abstract

**Objectives:**

The diagnosis of Bezold’s abscess can be challenging especially when craniofacial malformations imply facial and cervical morphological asymmetries. In addition, craniofacial malformations might predispose to the occurrence and atypical diffusion pathways of suppurative processes originating from abnormally developed temporal bone structures.

**Methods:**

A 30-year-old female presented with a left laterocervical swelling, worsening over time. The female was affected by Goldenhar syndrome. CT and MRI were performed.

**Results:**

CT revealed a dysmorphic os tympanicum and a deep cervical abscess in continuity with its cavity. Drainage of the cervical abscess was performed but a subsequent brain MRI detected a large cholesteatoma that was removed with left lateral petrosectomy.

**Conclusions:**

Radiology has a crucial role in the diagnosis and planning of the treatment of Bezold’s abscesses, particularly in syndromic patients. MRI, in this case, helped in diagnosing the presence of the cholesteatoma and consequently appropriately approach the surgical removal.

## Introduction

Oculo-auriculo-vertebral spectrum (Online Mendelian Inheritance in Male, 164210)^1^ is a rare congenital condition (incidence, 1: 3500–5600 live births)^2–5^ characterized by variable underdevelopment of craniofacial structures originating from the first and second pharyngeal arches. The resulting phenotype is considerably heterogeneous: common features are auricular abnormalities (such as microtia and aural atresia) and hemifacial microsomia.^6^ The most severe cases also present with eye or spine involvement (in these cases, oculo-auriculo-vertebral syndrome is also known as Goldenhar syndrome).^7^ The spectrum of abnormalities might encompass the central nervous system, cranial nerves and ipsilateral inner ear, internal carotid artery and salivary glands.^8–10^ Because of external ear malformation, these patients might be more vulnerable to regional infections, especially when a congenital cholesteatoma is present.^11^ In the most severe forms of facial asymmetry,^6^ local signs of inflammation, tympanic membrane bulging or hyperemia and otorrhea might not be evaluable or partly hindered by the altered anatomy of the temporal bone. Consequently, infectious processes might be easily overlooked, and severe complications might arise.^11^

Bezold’s abscess is a well-defined through extreme rare complication of mastoiditis^12^ due to erosion of the medial mastoid tip at the level of the digastric groove, determining the diffusion of the suppurative process into the posterior cervical space.^13^ First described in 1881 by the German otologist Friedrich Bezold, this complication has become less common most probably because of early management of mastoiditis with antibiotics as well as with modern imaging tools.^14^ Acute otitis media, cholesteatoma or necrotizing external otitis are the most common causes of mastoiditis associated with Bezold’s abscess.^14,15^ Typical clinical presentation is a floating laterocervical swelling frequently but not constantly accompanied by pain, fever, hearing loss, otorrhea and retroauricular swelling.^14^ Radiology has a crucial role in identifying the suppurative process, in delimiting its extension in the cervical spaces and in recognizing possible features, such as a coexistent cholesteatoma, mastoid bone erosions or peculiar anatomical characteristics that condition the subsequent surgical management.

Herein, we report on a patient affected by Goldenhar syndrome who developed Bezold’s abscess as a complication of an os tympanicum cholesteatoma.

## Clinical presentation and imaging findings

**Figure 1.**
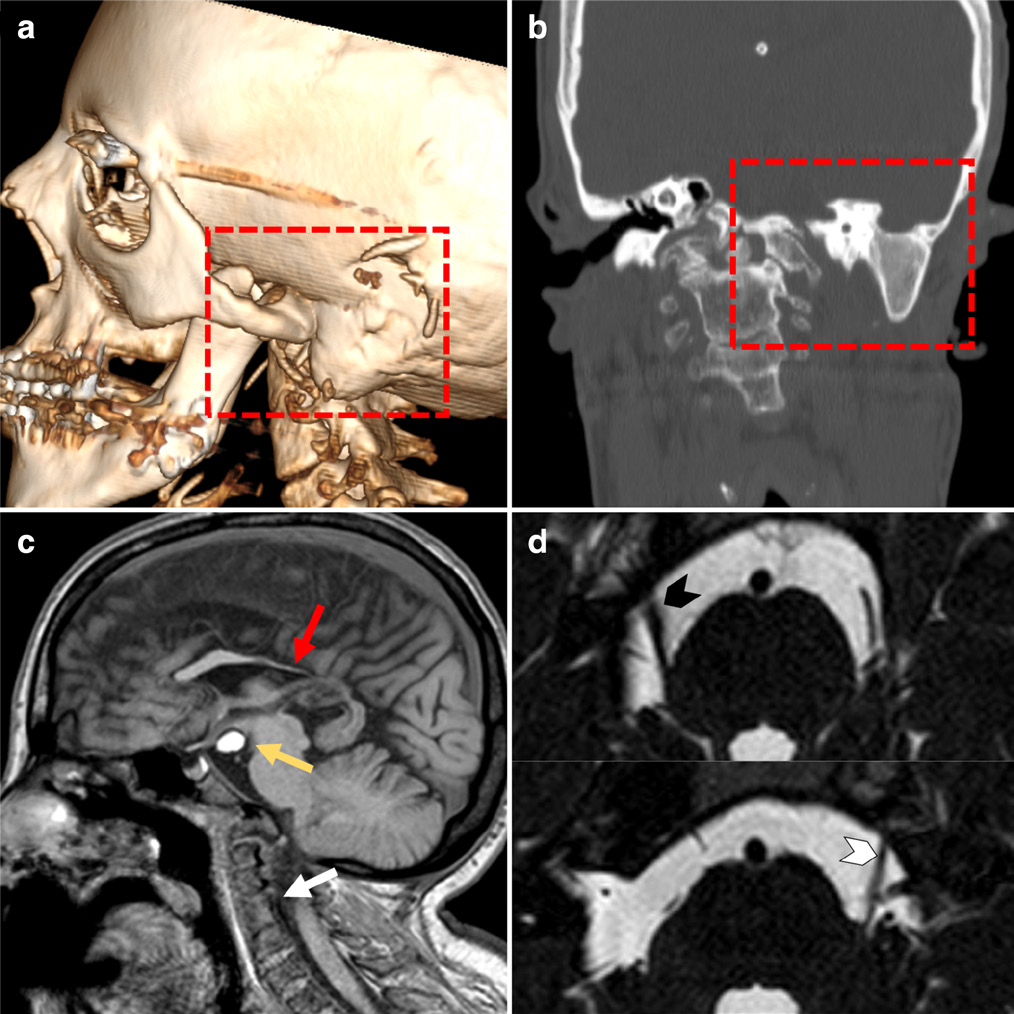
CT and brain MRI. (**a, **b) VRT and bone window coronal images showing aural atresia (red-dotted rectangle). The mastoid is sclerotic and it is not involved by the cholesteatoma. (c) sagittal T1 image showing concomitant brain and spine abnormalities encompassing midline lipoma of the floor of the III ventricle (yellow arrow), partial agenesia of the corpus callosum (red arrow), fusion of the upper cervical vertebras (white arrow). (d) high-resolution T2 axial images showing hypoplasia of the left (white arrowhead) trigeminal nerve (see right trigeminal nerve, black arrowhead, for comparison).

A 30-year-old female was admitted because of left laterocervical swelling, worsening over time despite protracted large spectrum antibiotic therapy. The female was affected by Goldenhar syndrome, with left craniofacial microsomia, aural atresia, microphthalmia and multiple cervical vertebral anomalies (Figure 1). Her medical history reported therapy-controlled epilepsy and peritoneal shunt for hydrocephalus since the early months of life. She had undergone multiple surgeries for jaw elongation and for aesthetic reconstruction of the left auricle, limited to the cutaneous and subcutaneous tissues, without involving the temporal bone.

**Figure 2.**
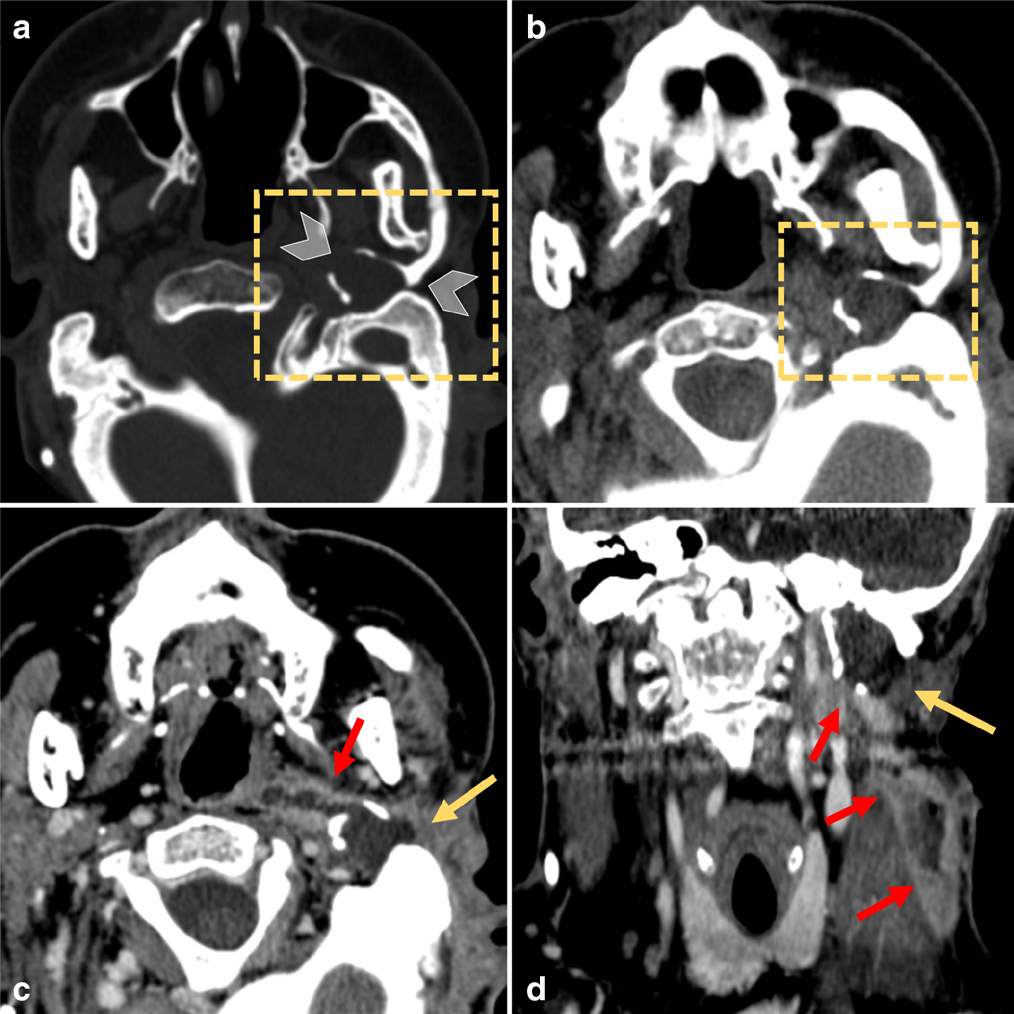
CT. Bone (a) and soft tissue (b) windows axial images showing the remodeling of the abnormal os tympanicum (dotted rectangle) filled with hypodense material. Wide cortical bone defects are visible in the lateral and posterior walls of the bone cavity (arrowheads). After contrast administration axial (c) and coronal (d) images show a Bezold’s abscess (red arrows) in continuity with the hypodense intraosseous material. Abscess walls enhanced markedly while the soft tissue in the os tympanicum (yellow arrows) showed minimal contrast enhancement. The mastoid is sclerotic, but it is not involved by the cholesteatoma.

At admission, head and neck CT (Figure 2) showed swelling of the left laterocervical soft tissues; the mastoid process was sclerotic but otherwise unremarkable. In contrast, a dysmorphic os tympanicum appeared enlarged, filled with hypodense tissue and presented with large cortical bone defects. After contrast medium administration, a deep cervical abscess appeared in continuity with the os tympanicum cavity.

**Figure 3.**
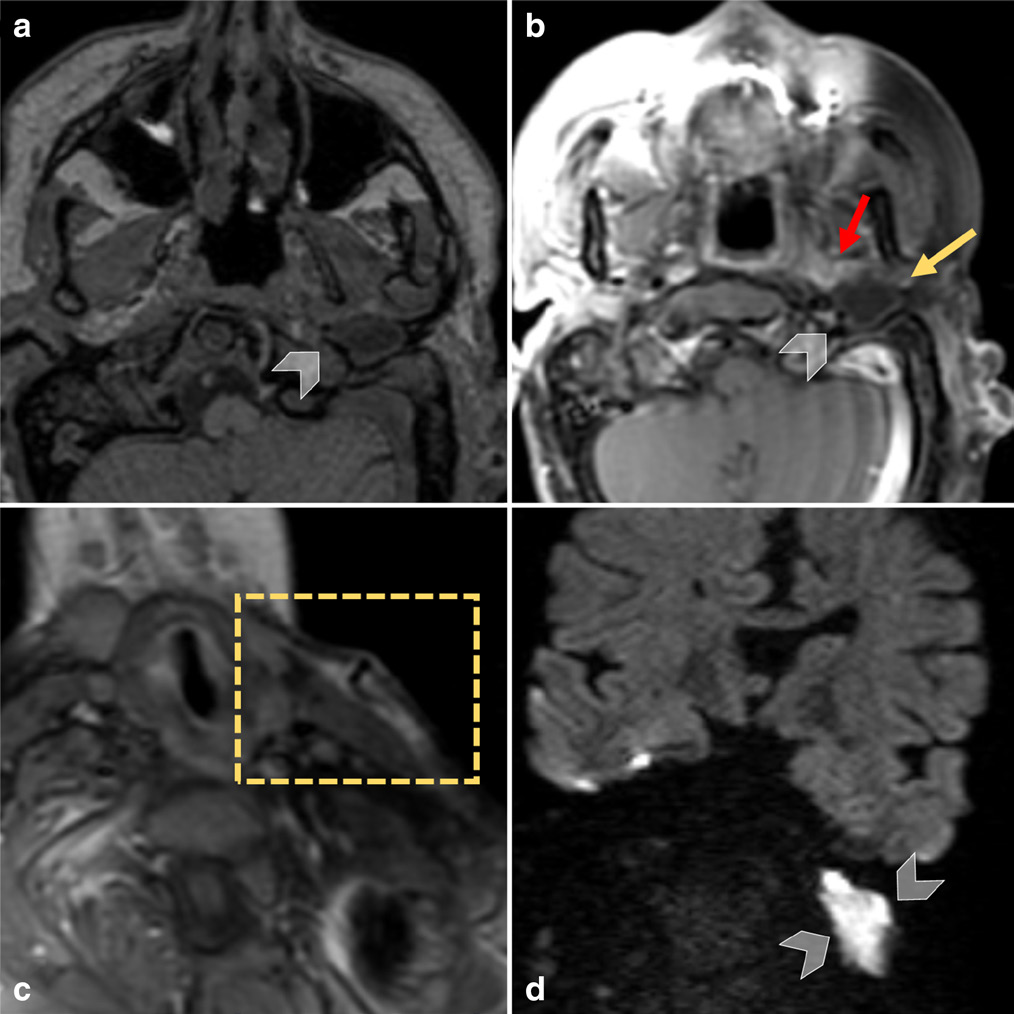
Brain MRI. Axial T1 images before (a) and after (b) contrast administration showing a very low intensity mass (arrowheads) in the os tympanicum with mild lateral peripheral enhancement (yellow arrow) and marked medial enhancement, consisting with abscess’s fistolous pathway (red arrow), as seen on CECT images. (c) Drainage in the laterocervical subcutaneous enhancing abscess (dotted rectangle). (d) Coronal diffusion-weighted images showing an hyperintense cholesteatoma (white arrowheads) in the bone cavity of the left os tympanicum.

After drainage of the laterocervical abscess, contrast-enhanced head and neck MRI (Figure 3) showed T2- and DWI-hyperintense (Echo-planar DWI, coronal acquisition, 4-mm slice thickness, b-value 1000 s mm^−2^) tissue inside the os tympanicum with milder peripheral contrast enhancement compared to the residual abscess’s walls (Figure 2) while apparent coefficient diffusion values were about 800 × 10^−6^ mm^2^ s^−1^. These findings were consistent with a large cholesteatoma. Besides, MRI also showed multiple brain abnormalities including midline lipoma, corpus callosum hypoplasia, vertebral abnormalities and left trigeminal hypoplasia (Figure 1). The left parotid gland was hypoplastic. The patient underwent left lateral petrosectomy with removal of the large cholesteatomatous mass, consistent with the imaging findings. The surgical field showed strong adherences of fibrous tissues and scars from previous surgeries in cutaneous and subcutaneous planes.

## Discussion

The definition of Bezold’s abscess clearly mentions the presence of an acute or chronic mastoiditis leading to a spread of the infectious suppurative process through the medial mastoid tip at the level of the digastric groove.^12,14^ However, the classic pathway described by Bezold might be strikingly subverted when craniofacial malformations are present. In our patient, the mastoid process was sclerotic but otherwise unremarkable. The infectious process, instead, involved the lateral petrous bone and spread into the posterior cervical space from the os tympanicum. Therefore, investigations of Bezold’s abscess in patients with craniofacial malformations should not focus exclusively on the mastoid and surgeons should carefully consider anatomic variations that might imply unexpected origins of infections and tailored surgical approaches.

Interestingly, our patient presented MRI hints of an underlying cholesteatoma that was eventually confirmed during surgery. Even though the patient previously had multiple surgeries to reconstruct the aesthetic of the external ear, the cholesteatoma could have also been congenital due to bullous remodelling of the os tympanic, the absence of direct surgical involvement of the temporal bone and the increased incidence of cholesteatoma in congenital microtia.^16^

Regardless of a congenital or acquired origin, cholesteatoma seems to be a remarkable predisposing factor for the development of an infective process in the petromastoid region, obstructing the normal osseous cavity airflow, favouring suppurative processes and their consequent spread towards the cervical region. Primary cholesteatoma or recurrent cholesteatoma after surgery have been reported in about one-third of Bezold’s abscess reports.^15^

These observations strengthen the diagnostic role of MRI, both in characterizing cervical soft tissue involvement and in recognizing the typical DWI hyperintensity of the cholesteatomatous tissue. A proper MR and CT imaging protocol appears to be pivotal, especially in patients with craniofacial malformations, where cholesteatomas are more common and often implicated in suppurative processes.^11^ In summary, heightened attention should be paid while treating malformed patients, especially because they have an increased risk of (congenital) cholesteatoma. They present a partially subverted anatomy, and their facial asymmetry might interfere with the identification of the laterocervical swelling leading to a delay in the diagnosis.^6^ As the association of cholesteatoma and mastoiditis might become an impending condition, in craniofacial malformation patients early imaging with bone CT and diffusion-weighted MRI sequences are pivotal for a prompt, tailored and adequate treatment.

## Learning points

1. The presence of craniofacial malformations might impair the early detection of a neck abscess
2. A Bezold’s abscess may arise from the os tympanicum
3. An underlying cholesteatoma might favour Bezold’s abscess formation.
4. For the best surgical management, the diagnostic work-up should include both bone CT and diffusion-weighted MRI.
